# Formability Prediction Using Machine Learning Combined with Process Design for High-Drawing-Ratio Aluminum Alloy Cups

**DOI:** 10.3390/ma17163991

**Published:** 2024-08-11

**Authors:** Yeong-Maw Hwang, Tsung-Han Ho, Yung-Fa Huang, Ching-Mu Chen

**Affiliations:** 1 Department of Mechanical and Electro-Mechanical Engineering, National Sun Yat-sen University, Kaohsiung 80424, Taiwan; ymhwang@mail.nsysu.edu.tw (Y.-M.H.); rf30526@gmail.com (T.-H.H.); 2 Department of Information and Communication Engineering, Chaoyang University of Technology, Taichung 413310, Taiwan; 3 Department of Electrical Engineering, National Penghu University of Science and Technology, Magong 88046, Taiwan; t20136@gms.npu.edu.tw

**Keywords:** deep drawing, machine learning, aluminum alloy A7075, finite element analysis

## Abstract

Deep drawing has been practiced in various manufacturing industries for many years. With the aid of stamping equipment, materials are sheared to different shapes and dimensions for users. Meanwhile, through artificial intelligence (AI) training, machines can make decisions or perform various functions. The aim of this study is to discuss the geometric and process parameters for A7075 in deep drawing and derive the formable regions of sound products for different forming parameters. Four parameters—forming temperature, punch speed, blank diameter and thickness—are used to investigate their effects on the forming results. Through finite element simulation, a database is established and used for machine learning (ML) training and validation to derive an AI prediction model. Importing the forming parameters into this prediction model can obtain the forming results rapidly. To validate the formable regions of sound products, several experiments are conducted and the results are compared with the prediction results to verify the feasibility of applying ML to deep drawing processes of aluminum alloy A7075 and the reliability of the AI prediction model.

## 1. Introduction

As a widely used metal forming technology, deep drawing processes have been applied to manufacturing circular or square cups in various industries for many years. With the help of stamping equipment, blanks are sheared, bent, and shaped into desired geometries and dimensions. Artificial intelligence (AI) is an emerging field of science and technology development. The purpose of AI is to allow computers to learn like humans, and through training with a database to make decisions or perform various functions. The objective of this study is to explore the influence of forming temperature, punch speed, blank diameter, and blank thickness on the forming results using finite element simulations with the variation of the four parameters and obtain a possible forming range for sound-drawn products in A7075 deep drawing processes using machine learning (ML) algorithm. A dataset for formable ranges of the parameters is constructed from ML training and verification. An AI prediction model is also established. Inputting the forming parameters, the predicted forming results can be obtained quickly. Finally, deep drawing experiments are conducted and the results obtained are used to verify the predicted formable regions of sound products and the proposed AI model.

Deep drawing is a long-established technology. Using a stamping machine, a circular sheet can be formed into circular cups or more complex shape parts used in automotive or aircraft industries [1]. Due to long-term development, deep drawing and stamping have become a stable and well-known technology. However, these methods are limited to forming ductile metal materials. For some brittle metal materials with low formability and high hardness, such as aluminum alloy A7075, cracking or fractures are likely to occur at the cup corner during deep drawing processes. To raise the formability of the products, an elevated temperature is usually used, and other forming parameters have to be adjusted appropriately. It is necessary to obtain the formable ranges for various forming parameters. The finite element analysis has been widely applied to construct a comprehensive model for many different cases. Nevertheless, a lot of time is needed to complete all the simulations with various cases. Therefore, a new approach based on ML is proposed to replace the traditional finite element simulation-based method. The ML system learns from input data and improves the output results by adjusting internal weights. After training, it can be used to predict results or assist decision-making. After learning, the predicted forming results can be obtained quickly under different forming conditions by inputting relevant information later. This approach can save time and cost in developing various deep-drawn cup products.

Regarding the mechanical properties of aluminum alloy A7075, Tajally and Emadoddin [2] and Cerri et al. [3] investigated the mechanics and anisotropic behavior of A7075 plates. The former conducted annealing of A7075 plates to reduce the yield strength and hardness and to improve its formability. A series of experiments verified that annealing at 350~400 °C has obvious effects. The latter conducted high-temperature torsion testing. As the temperature increases, the ductility between 250 °C and 300 °C increases due to changes in the microstructure. In view of the influence of various forming parameters of deep drawing, Colgan and Monaghan [1] examined some forming factors influencing the deep drawing process, utilizing an experimental rig design and statistical analysis. The parameters include the punch and die radii, the punch velocity, clamping force, friction, and drawing depth. From finite element analysis and experimental results, it seems that the punch/die radii have the greatest effect on the thickness of the deformed mild steel cups compared to blank-holder force or friction. Chen et al. [4] used finite element analysis to investigate the effects of the gap between the punch and die, the corner radius of the punch, and the corner radius of the die shoulder in deep drawing with a circular and square die design. They found that the defects of cracks occurred due to excessive stretching of the blank. To avoid cracking to improve the formability, the die geometrical dimensions and forming parameters have to be set approximately. Gowtham et al. [5] investigated the effects of the die radius on deep drawing results and found that reducing the die radius can cause stretch marks on the blank and uneven height of the cup.

Concerning the studies on AI, a few papers have been published to examine network algorithms to combine metal-forming technology with ML. For example, Liu et al. [6] used a long short-term memory (LSTM) network to predict bearing failure and fracture and proposed a new LSTM model. This model combines the advantages of an LSTM network and statistical analysis to predict aviation engine bearing failure. The results show that this method has higher accuracy than recurrent neural network (RNN), support vector machine (SVM), and LSTM, thereby improving the prediction accuracy of bearing performance degradation trend and remaining service life. ML facilitates computers to read and interpret from the previously present data automatically and makes use of multiple algorithms to build models, mathematical in nature, and then makes predictions for the new data using the past data and knowledge. Lately, it has been adopted for text detection, hate speech detection, recommender system, face detection, and more. In [7], the aspects concerning ML algorithms, K-nearest neighbor (KNN), genetic algorithm (GA), SVM, decision tree (DT), and LSTM network have been investigated.

In [8], the authors calculated the accuracy of ML algorithms for predicting heart disease, with KNN, DT, linear regression, and SVM by using the University of California, Irvine (UCI) repository dataset for training and testing. In [9], the previous works proposed a hybrid traffic classification method based on ML using the packet-multilayer perceptron (P-MLP) model and majority voting method to effectively classify the encrypted traffic in the network. In previous research [10], a FaceNet training method is used to train the mask face recognition (MFR) model using migration learning combined with a CNN model and a fully connected SoftMax output classifier to dynamically update the optimizer’s learning rate (LR). The proposed ML models combined with fingerprinting for indoor localization using channel side information (CSI) [11]. Experiments were conducted on the positioning accuracy performance of RF and backpropagation neural network (BPNN) ML models using received signal strength indicator (RSSI) and CSI information, respectively.

Shinomiya et al. [12] used ML to control the movement of a slider and applied a convolutional neural network (CNN) with consideration of the elastic strain of the punch to predict the quality of the extruded products. Using this intelligent slider motion control, the defects can be prevented, and sound products can be obtained. Accordingly, the defect rate can be reduced significantly. Crystal plasticity analysis in sheet metal forming consumes a lot of calculation time. Cancemi et al. [13] proposed deep learning (DL) in the investigation of the safety behavior of nuclear plant items. The proposed innovative methodology is based on an unsupervised neural network (NN) to predict potential anomalies in the cooling system of a pressurized water reactor. Yamanaka [14] used ML models to replace complicated formula calculations and used simpler dimensionality reduction models to calculate the data from sheet-forming experiments. Thus, the efficiency of sheet metal forming simulations is improved by combining with the ML learning models.

Due to the shortage of manpower, many manufacturing companies are developing their production toward automation and intelligence. Therefore, in recent years, many companies introduced metal-forming technology combined with AI. In this paper, a new approach based on ML is proposed to predict the formable regions of a sound product for various forming parameters in the deep drawing process of an aluminum ally A7075 circular cup. By this method, the development cycle of a newly drawn product can be shortened and the production efficiency can be improved.

## 2. Finite Element Simulations

### 2.1. Geometric Configurations in the Deep Drawing Process

DEFORM (v11.0.2, Scientific Forming Technologies Corporation (SFTC), Columbus, OH, USA) [15], which is a finite element simulation software, was used in this paper for a series of simulations. It was used to carry out deep drawing simulations to analyze the plastic deformation and heat transfer of the metal material and exhibit the simulation results of drawing force and product geometries.

**Figure 1:**
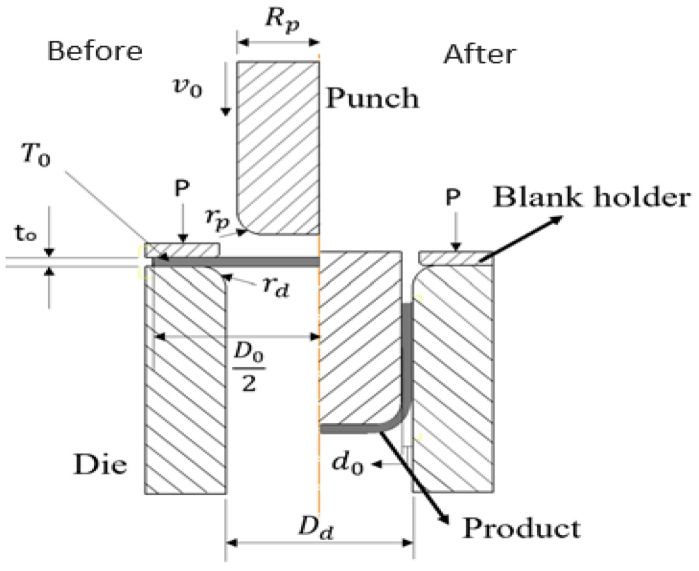
Schematic diagrams of before and after deep drawing forming processes.

The schematic diagrams of before and after deep drawing forming processes are shown in Figure 1, and the relevant geometrical dimensions and forming parameters used in finite element simulations are shown in Table 1. The simulation objects are mainly composed of four parts: a punch, a blank holder, a blank, and a die. During the simulation process, the punch moves downward at a constant speed. After contacting the blank material, punch force is applied to bend the blank downward and press it into the die. During this period, a blank holder is used to compress the blank. In the actual experiments, a spring is used as the source of the compression force; thus, the compression force is set as the function of punch stroke to ensure the simulation conditions are the same as those in the experiments. The circular blank material is aluminum alloy A7075. Initially, the blank is placed on the top of the die and compressed by the blank holder. It is bent by the downward punch and drawn into the die cavity to form a cup-shaped product. The gap between the punch and the die is an important geometric parameter, which is usually set as 1.1–1.3 times the blank thickness. If the gap is smaller, a longer cup can be obtained, but it may result in breakage at the cup wall. If the gap is too large, wrinkles or uneven material stretching may occur. In this study, the die gap is set at 2.2 mm, 1.1 times the blank thickness. The blank material of aluminum alloy A7075 is set as an elastoplastic body, whereas the punch, die and blank holder are regarded as rigid bodies for saving the simulation time. The material properties of aluminum alloy A7075 used in the finite element simulations are shown in Table 2.

### 2.2. Convergence Analysis for Finite Element Simulations of Deep Drawing Process

**Figure 2:**
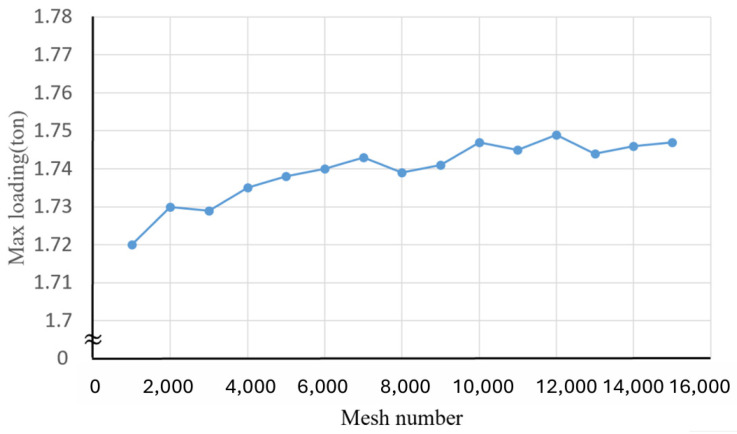
Effects of the element number on maximal load variation in the deep drawing process.

To discuss the maximal deviation of the simulation results, convergence analysis of maximal load variation in deep drawing processes was implemented. The effects of the total element number on the maximal load variation in deep drawing processes are shown in Figure 2. Clearly, as the element number increases from 1000 elements (8-layer elements in the thickness direction) to 10,000 elements (25-layer elements in the thickness direction), the maximal load converges gradually from 1.72 tons to 1.74 tons and the maximal variations decrease by 0.5% as the element number exceeds 10,000. Accordingly, an element number of 10,000 is adopted in the subsequent finite element simulations.

### 2.3. Compression Tests of A7075

**Figure 3:**
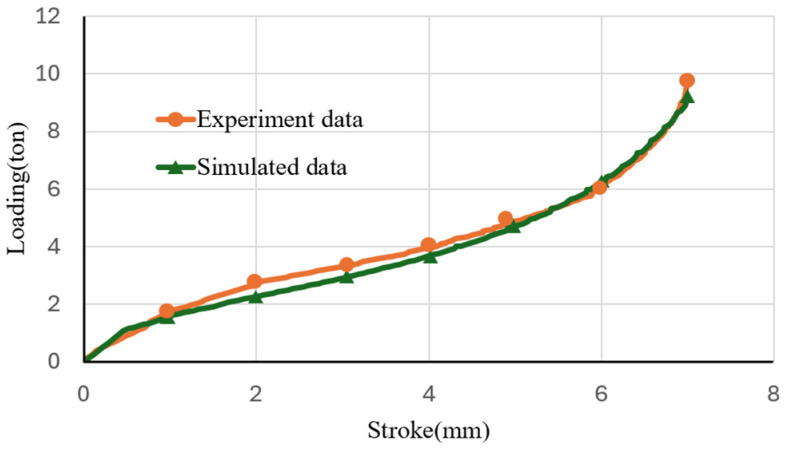
Comparisons of load-stroke curves between simulations and compression tests.

In deep drawing processes, the blank material of A7075 undergoes plastic deformation. Thus, compression tests of aluminum alloy A7075 using a 160-ton servo press were conducted to obtain its flow stresses. In compression test experiments, the load and displacement of the upper die were recorded. The load is divided by the cross-section area of the specimen to obtain engineering stress and the top die displacement is divided by the initial length of the specimen to obtain engineering strain. Engineering stress and engineering strain can be converted into true stress $\sigma_{t}$ and true strain $\varepsilon_{t}$ through the equations by

(1) $$\sigma_{t}=\sigma_{e}\left(1+\varepsilon_{e}\right),$$

and

(2) $$\varepsilon_{t}=ln(1+\varepsilon_{e})$$

respectively, where $\sigma_{e}$ is the engineering stress and $\varepsilon_{e}$ is the engineering strain [17]. The obtained A7075 flow stress curves are used in the finite element simulations. To ensure the accuracy of the flow stress obtained, finite element simulations of compression tests using the calculated flow stress were conducted. The comparisons between simulated and experimental loads are shown in Figure 3. The maximal difference between the two loads is about 10%, which validates the flow stress curve used in the finite element simulations. Compression tests at different temperatures were conducted. The flow stresses at different temperatures were input into DEFORM for the simulations at different forming temperatures. Heat conduction among the punch, blank and die was set. The related coefficients are given in Table 2.

The critical fracture value of the material varies with the forming temperature. Normalized Cockcroft–Latham ductile fracture criteria [16] are used to calculate the fracture value of each point in the forming blank. During deformation, as the damage value at any point inside the blank is larger than the critical damage value, the point or element will disappear, which means a crack or fracture may occur. The critical damage values of A7075 at different temperatures shown in Table 3 were obtained by comparing the simulated compression tests with the actual compression tests. A7075 has tiny holes in the internal crystal lattice at higher temperatures, thus, the critical fracture values decrease slightly at higher temperatures [18].

### 2.4. Simulation Results and Discussion

A series of static-implicit finite element simulations with variations of the four forming parameters: forming temperature, punch speed, blank thickness, and blank diameter were conducted. Several levels for each parameter are chosen for the finite element simulations. The values or levels for the four forming parameters are listed in Table 4.

**Figure 4:**
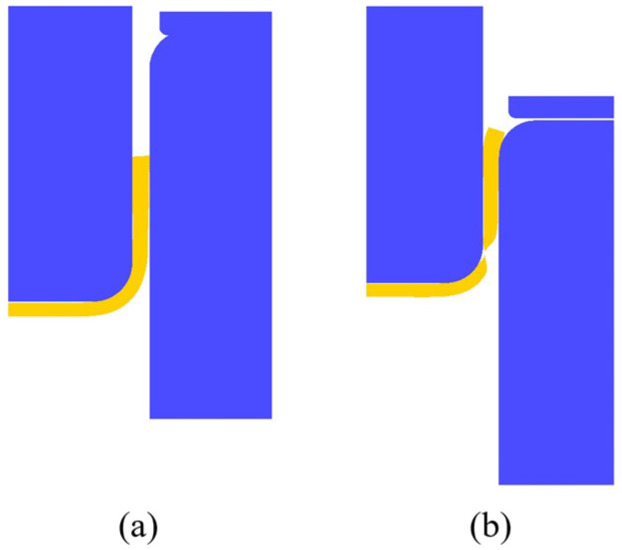
Simulation results with different temperatures: (**a**) 250 °C and (**b**) 375 °C.

In case 1, the punch speed, blank diameter, and thickness are fixed at 12.1 mm/s, 57.5 mm, and 2.0 mm, respectively, and finite element simulations with variations of forming temperature were conducted. The simulation results with temperatures of 250 **°**C and 375 **°**C are shown in Figure 4a and Figure 4b, respectively, where the product is shown by light yellow color for easily distinguished. Clearly, a sound product was obtained at a temperature of 250 **°**C, whereas fracture occurred around the corner of the cup at the temperature of 375 **°**C. From a series of simulation results, it is known that at either too low or too high temperatures necking or fracture occurred in the product. The blank at lower temperatures is subjected to greater stress during deformation and is prone to cracking at the fillets or corners of the deep-drawn cup. Because of smaller critical damage values at higher temperatures, failure or cracks are easier to occur during the drawing process. Therefore, deep drawing processes should be conducted within a certain temperature range for better-quality products.

**Figure 5:**
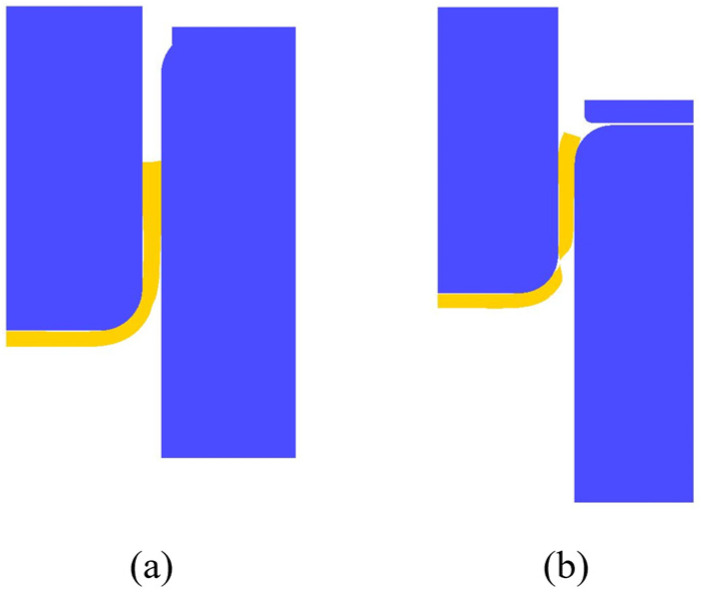
Simulation results with different punch speeds: (**a**) 36.3 mm/s and (**b**)12.1 mm/s.

In case 2, the forming temperature, blank diameter, and thickness are fixed at 375 **°**C, 57.5 mm, and 2.0 mm, respectively, and finite element simulations with variations of punch speed were conducted. The simulation results with punch speeds of 36.3 mm/s and 12.1 mm/s are shown in Figure 5a and Figure 5b, respectively. Clearly, a sound product was obtained at a punch speed of 36.3 mm/s, whereas a fracture occurred around the corner of the cup at a punch speed of 12.1 mm/s. From a series of simulation results, it is known that at a faster punch speed, a sound-drawn cup can be obtained, whereas, at a slower punch speed the blank is easier to break during the drawing process. That is probably because a slower punch speed may limit the plastic deformation of the blank and a too low strain rate may result in uneven product surfaces.

**Figure 6:**
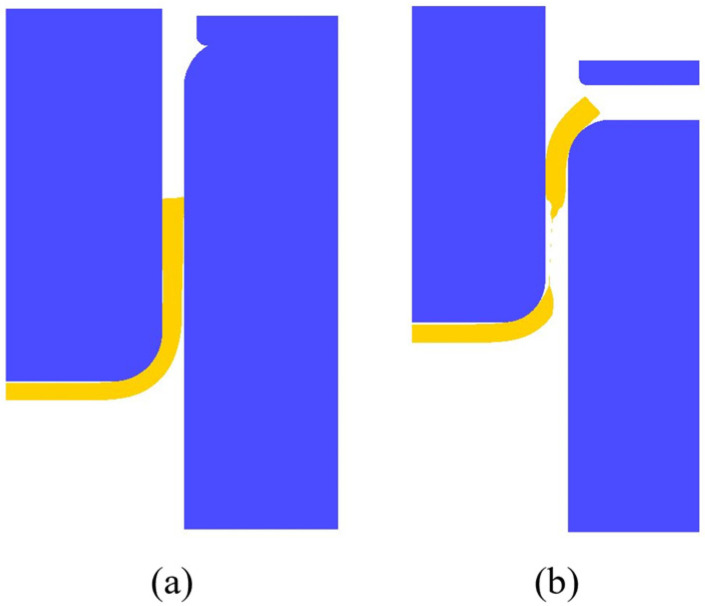
Simulation results with different blank thicknesses of (**a**) 2.0 mm and (**b**) 2.5 mm.

The blank diameter affects greatly the drawing ratio of the deep-drawn cup. The limited drawing ratio is expressed by the following:

(3) $$LDR=\frac{D_{m}}{D_{p}}\;$$

where $D_{p}$ is the punch diameter and $D_{m}$ is the blank diameter [19]. In case 3, the forming temperature, punch speed, and blank diameter are fixed at 375 °C, 29.7 mm/s, and 65 mm, respectively, and finite element simulations with variations of blank thickness were conducted. The simulation results with blank thicknesses of 2.0 mm and 2.5 mm are shown in Figure 6a and Figure 6b, respectively, where the product is shown by yellow color for easily distinguished. Clearly, a sound product was obtained at a blank thickness of 2.0 mm, whereas fracture occurred at a blank thickness of 2.5 mm. From a series of simulation results, it is known that it is difficult to form a too-thick or too-thin blank. For thicker blanks, it is difficult to bend the blank at the round corner, which results in a bad cup shape. For thinner blank, although it is easier to bend at the round corner, however, its strength decreases, which may lead to cracking at the cup wall. Therefore, the blank thickness should be within a range to achieve better-forming results.

## 3. Machine Learning Classifier

A classifier is an ML model that assigns data points to different categories or labels, using supervised learning methods to learn labeled data to predict the category of subsequent data [20]. With the development of modern AI, its advantages are gradually valued by the manufacturing industry. In terms of improving production efficiency, AI’s high degree of learning and efficient computing power can make the most appropriate decisions quickly, or by establishing a dataset for AI to learn, subsequent data can be processed in various tasks [12]. 

This study uses the finite element software simulation results explained in the previous section as input data to write a prediction model and create an input dataset, allowing the model to learn the input dataset, predict subsequent inputs, and obtain the forming range of aluminum alloy 7075. In this study, the classifier function in ML is used to input data such as material temperature, material diameter, forming speed, and other parameters and forming results to classify, thereby predicting whether the input forming parameters can be successfully formed in the future. The classifiers used in this study can be divided into two categories: weak classifiers and integrated classifiers. Weak classifiers are mainly composed of a single classifier model. Weak classifiers are faster in learning speed but have lower learning rates or relatively poor accuracy. 

The weak classifiers used in this study include Logistic regression, KNN, SVM, DT, etc. Integrated learning mainly consists of multiple or multiple weak classifiers, which are systematically integrated, and then the final classification prediction is obtained through weighted calculation or voting calculation. Compared with weak classifiers, due to the use of multiple weak classifiers for combined learning, the accuracy and learning effect of integrated learning classifiers are usually better than those of general weak classifiers. 

AdaBoost, the full name of adaptive boosting, also known as adaptive enhancement, is an integrated learning classifier that uses the Boosting concept [20]. The basic concept of Boosting is to combine multiple weak classifiers to form a strong classifier with a strong classification effect. The main idea is to improve prediction results through iteration. Boosting uses a weighting method to process the original data. The data of each training input set is given an initial weight by the model. In each round, a new weak classifier is used to train the training set, and each classifier is also given initial weights. After each round of learning, the data are divided into two categories: correctly classified and incorrectly classified. The weights of the two data types are updated, increasing the weight of the incorrectly classified data and reducing the weight of the correctly classified data so that the model can respond to the incorrectly classified data. The data are learned again, and the updated weights are given to the next round of classifiers for re-training. 

**Figure 7:**
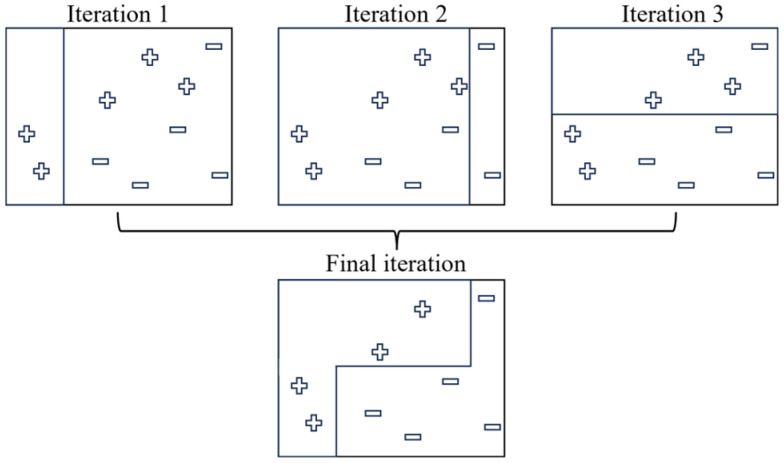
AdaBoost algorithm iteration structure.

Since the efficiency and accuracy of each classifier are different, all classifiers need to be integrated at the end of all training. At this time, the weight of the classifier with a higher error rate and poor accuracy is reduced, while the weight of the classifier with better performance and poor accuracy is reduced. The weight of higher classifiers increases, and finally, the updated weights of all classifiers and their corresponding classification results are statistically calculated to obtain the prediction results of the Boosting model. The structure diagram of AdaBoost is shown in Figure 7 [21]. After iterative training of the classifier, the overall model is gradually improved, and finally, excellent prediction results are obtained.

In terms of programming, Python is used to write research-related programs and establish a virtual environment for running classification prediction models. The input data learned by the program are the four forming parameters and forming results set in the Deform simulation. The four parameters are forming temperature, punch speed, material diameter, and material thickness. A total of 624 pieces of the input dataset are recorded for model training, as shown in Table 5, where the forming results are represented by 0 and 1, where 1 represents the material being formed smoothly, and 0 represents material rupture.

**Figure 8:**
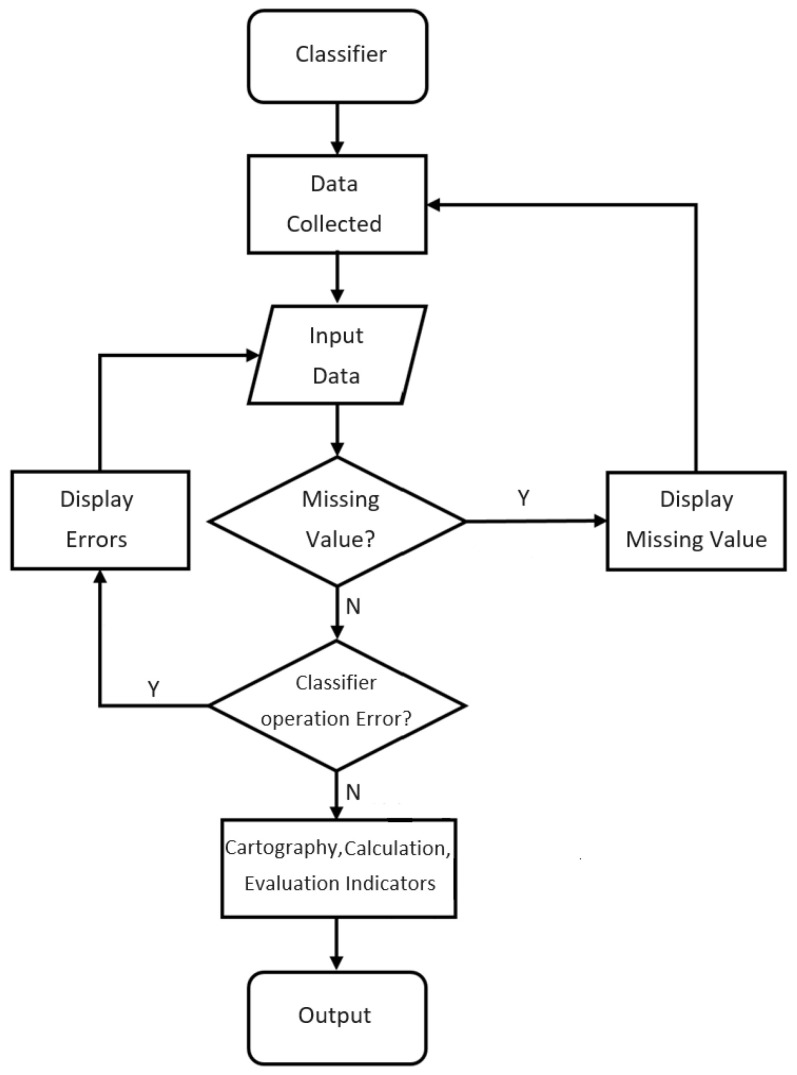
Flow chart for the classification prediction model.

Input the dataset into the program and use it. The data are divided into two categories: training and test sets, with the number of records being 8:2. Then, write the classifier used in the study. The operation flow chart of the program is shown in Figure 8. The input data are imported into the specified folder at the model’s front end. It is necessary to check whether the input dataset has missing values or other incorrect data formats before it can be input into the classifier for internal learning. Otherwise, it needs to be re-entered. Confirm the content of the dataset. The program will pop up an error message if an error occurs during the classifier learning process. You must reconfirm the input dataset or modify the classification model to avoid program code or logic errors.

After the input dataset is confirmed to be correct, the model will be divided into training and test datasets. In ML, to evaluate the accuracy of the prediction model, the model will use the data of the training set to learn and then predict the output of the test set. The accuracy is obtained by comparison. The ratio of training and test sets is 8:2. After obtaining the test set, a program must be set up to distinguish the data used for prediction and the output result data. In this study, the four data types were used for prediction. The parameter factor, the output result data, is the material forming result. After the data are learned by the classifier, the results of model learning need to be tested.

The trained model predicts the output of the test set, and the results will be compared with the original correct answers. Evaluation indicators such as accuracy and error will be recorded. After training and testing, the model will summarize and export the model evaluation indicators of all classifiers and present the prediction results in charts, drawings, etc., to facilitate the researchers’ interpretation. Model evaluation indicators can be used to determine which classifier has the best performance and the most accurate prediction results, and the classifier with the best results can be selected as a classification prediction system. In the future, one only needs to enter relevant forming parameters, and the model will provide prediction results for reference.

**Figure 9:**
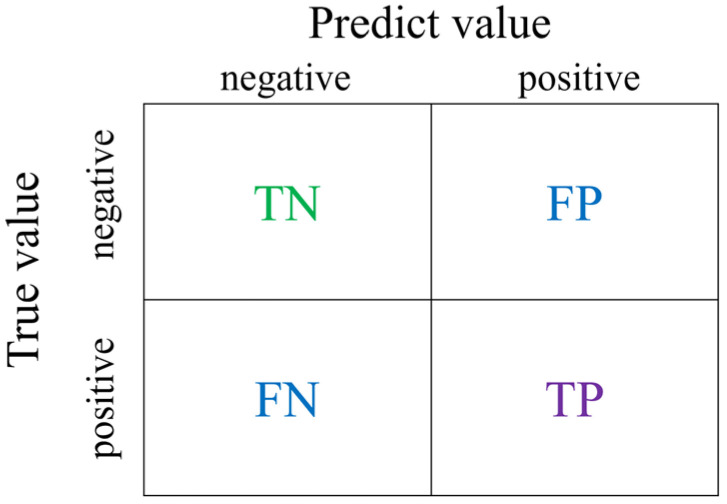
**C**onfusion matrix for the classification prediction model.

The confusion matrix is a 2 × 2 matrix. The horizontal and vertical axes represent the actual and predicted results, respectively. The four blocks of the matrix represent different meanings, as shown in Figure 9 [22].

**Figure 10:**
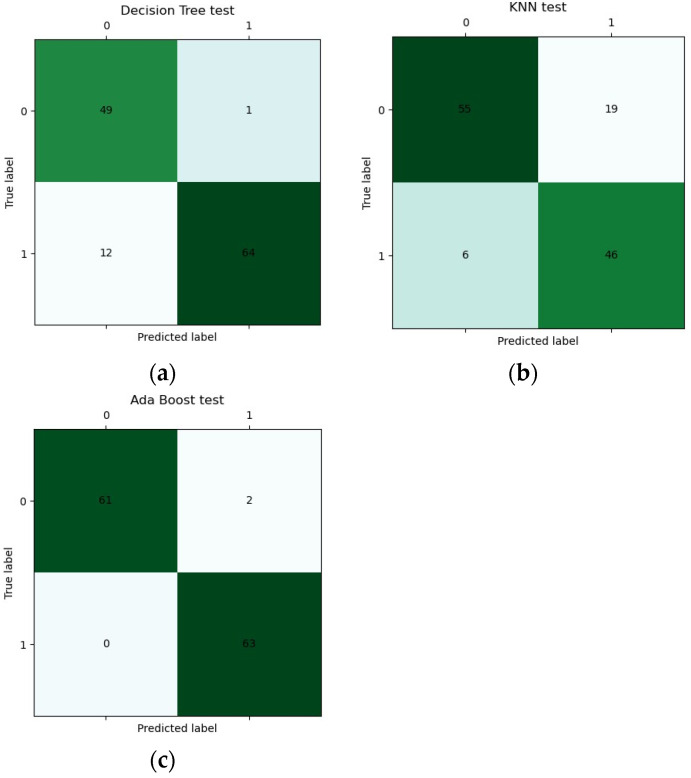
Some confusion matrices of classifiers: (**a**) DT, (**b**) KNN, and (**c**) AdaBoost.

Some results of the confusion matrix of classifiers are shown in Figure 10, where the dark color is used for highlighting the values. From Figure 10a, it is observed that the weak classifier of DT suffers a higher FN with 12. From Figure 10b, it is observed that the weak classifier of KNN suffers higher FP with 19. However, From Figure 10c, it is observed that there are lower FP with 2 and FN with 0 for the strong classifier of AdaBoost.

Other evaluation indicators can be derived from the confusion matrix, and various model functions can be analyzed. The accuracy rate indicates the accuracy of the model’s incorrect prediction. The calculation formula is shown as follows:

(4) $$Accuracy=\frac{TP+TN}{TP+FP+FN+TN}.$$

The highest accuracy may not always mean that the model’s prediction effect is the best, so it needs to be combined with other evaluations. Indicators are discussed together. The precision rate represents the model’s accuracy in predicting positive examples. The calculation formula is shown as follows:

(5) $$Precision=\frac{TP}{TP+FP}$$

where the higher the ratio of TP, the higher the model’s accuracy. A high accuracy rate means that the model is less likely to make mistakes when its prediction is positive. The recall rate represents the rate of correct samples being classified as positive. The calculation formula is shown as follows:

(6) $$recall=\frac{TP}{TP+FN}.$$

**Figure 11:**
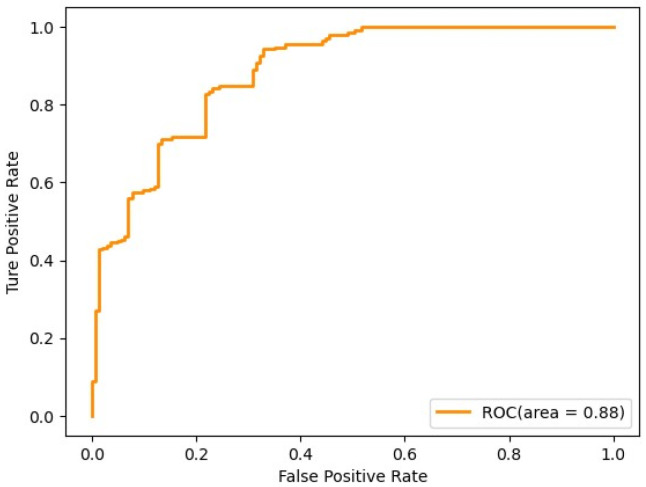
ROC curve diagram.

A high recall rate means that the higher the proportion of correct samples that are judged as positive in the model, the less likely it is that the correct sample will be judged as wrong. The calculation formula of the *F*1 score is shown as follows:

(7) $$F1score=2\frac{precision\times recall}{precision+recall}$$

which requires using two widely referenced values, precision and recall because they can reflect the algorithm’s accuracy. The receiver operating characteristic curve (ROC curve) is a visual accuracy indicator, as shown in Figure 11. The ROC curve’s horizontal and vertical axes represent the model’s false positive rate (FPR) and true positive rate (TPR). The farther the curve result deviates from the diagonal, the higher the accuracy. The larger the area under the curve (AUC, Area under the curve), the higher the accuracy and the better the model performance. The AUC value can compare the performance of different models, and models with larger AUC have better classification performance [23].

The numerical values of the evaluation indicators can be used to determine which classifier model is better. Compare the accuracy, precision, recall, F1 score, and area under the ROC curve AUC, as shown in Table 6. The closer the values of these five indicators are to 1, the better the performance and effect of the model. From the values in the table, we know that the first 6 rows are the results of weak classifiers. In terms of accuracy, some weak classifiers have higher accuracy. The precision and recall rate values are also the same as the accuracy rates. The performance of weak classifiers is mostly between 0.84 and 0.94. The prediction effect needs to be strengthened. Since the values of the precision rate and recall rate also affect the performance, the F1 score’s value of the F1 score also fluctuates wildly. The value of AUC reflects the predictive value of the model.

In Table 6, the evaluation index results of the integrated learning classifiers are shown in the 7th to 11th rows. From the numerical comparison, the integrated learning’s accuracy, precision, and recall have greater progress than the weaker classifiers. The accuracy, precision, and recall rate even have a performance of 1, which improves prediction accuracy. The closer the AUC is to 1, the higher the prediction value of the model. Therefore, the ensemble learning classifier has a better prediction effect and higher classification accuracy than the weak classifier. The model has better predictive value, so this study will use an integrated learning classifier as the main architecture of the classification prediction model, in which the Ada Boost classifier is used to predict the material formability range of aluminum alloy A7075.

**Figure 12:**
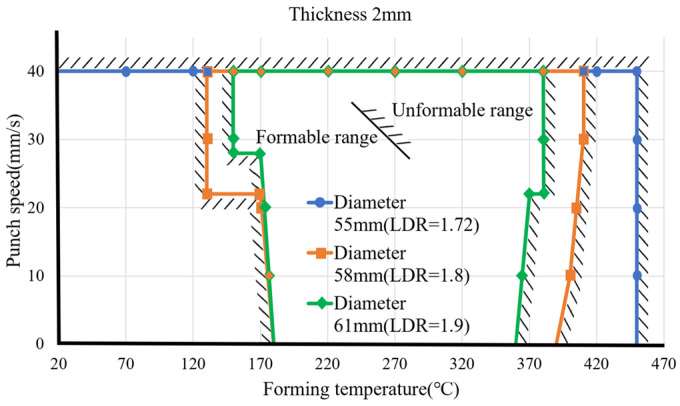
Formable ranges for different blank diameters.

After the model completes learning, the subsequent input of forming parameters can predict the results. From this, different forming parameters can be input to obtain the formable range of the material. The material thickness is fixed at 2 mm, and the forming ranges under different forming temperatures, punch speeds, and material diameters are compared, as shown in Figure 12. The three lines in the figure represent the three diameters of aluminum alloy 7075 discs, respectively, with diameters of 55, 58, and 61 mm. The area under the curve is the range in which the material can be successfully formed. The forming range of the material with a diameter of 61 mm is compared to the diameters of 55 and 58 mm. The range is small and difficult to form at low or too-high temperatures. The forming range of a diameter of 61 mm is approximately above 180 °C and below 350 °C. Forming in this range will have better results. The forming range of a material with a diameter of 58 mm is larger than that of 61 mm. However, it is also difficult to form at room (or lower) temperatures due to excessive material stress. Too high temperatures will also cause poor forming effects. Therefore, the formable range is from approximately 170 °C to 380 °C. The forming range of the material with a diameter of 55 mm is relatively loose, and the forming range can be smoothly formed from 250 °C to as high as 450 °C. Since the deep extension cup formed by the material disc with a diameter of 55 mm has a relatively low extension, the specimen material has a relatively low extension. The deformation is not large, and the forming conditions are relatively loose, so the impact of temperature and stamping speed is small.

**Figure 13:**
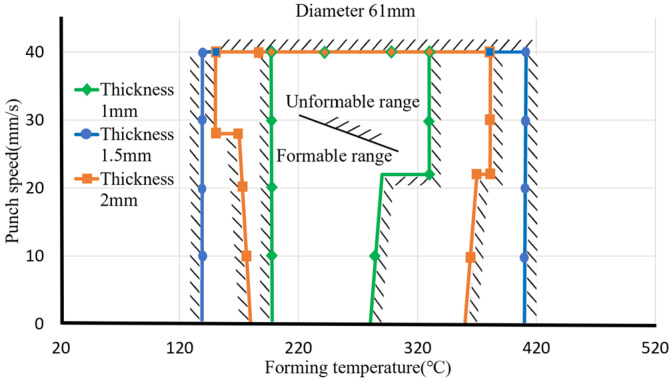
Formable ranges for different blank thicknesses.

The material diameter is controlled to 61 mm and the stretch ratio to 1.9. We change the material thickness, forming temperature, and punch speed for comparison, as shown in Figure 13. The three lines represent the material forming range with material thicknesses of 1, 1.5, and 2 mm, respectively. The forming curve range of a material with a thickness of 1 mm is small. The forming temperature is about 170 °C to 270 °C. It can be formed smoothly. After 270 °C, the punch speed needs to be controlled. It cannot be formed after 340 °C. Therefore, the forming range of a 1 mm material is more suitable for forming between 200 °C and 250 °C. The forming range of a material with a thickness of 1.5 mm is larger than that of a material with a thickness of 1 mm. The formable temperature range falls between 140 °C and 410 °C. The blank is not easy to form at a too-low temperature, so deep processing should be carried out between 170 °C and 370 °C. The extension-forming effect is better. The forming range of a material with a thickness of 2 mm is larger than that of 1 mm but smaller than that of 1.5 mm. The forming temperature range is about 170 °C to 370 °C. Forming at low or high temperatures is difficult, so it is suitable. The forming temperature is about 200 °C to 350 °C.

**Figure 14:**
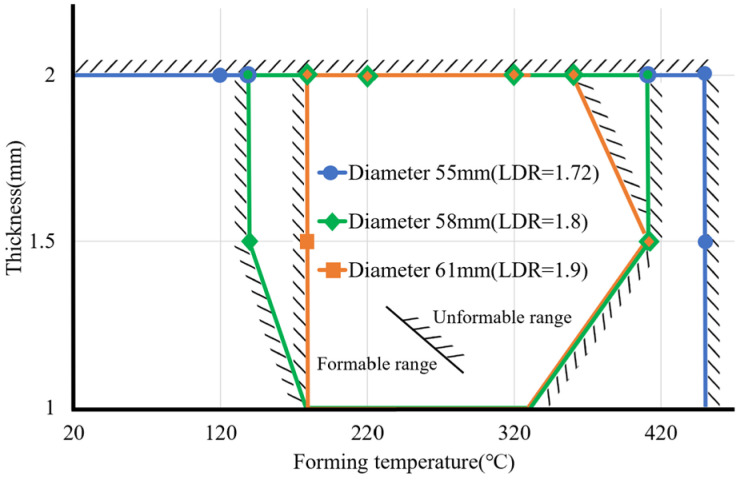
Formable ranges of temperature for different blank thicknesses and diameters.

To sum up, under the conditions of the same material diameter and the same draw ratio, the suitable thickness of the extension cup falls within a range rather than excessively increasing or reducing the thickness. If you want to produce thinner or thicker plates for a deep extension cup, the extension ratio of the extension cup needs to be reduced; that is, the material diameter can be reduced to form effectively. From the previous results, it can be concluded that the three parameters—forming temperature, material diameter, and material thickness—will have a greater impact on the forming of deep extension cups, while the impact of punch speed is relatively small. The punch speed is fixed at 24 mm/s, and the effects of the other three parameters are compared, as shown in Figure 14.

As the blank diameter increases or the draw ratio increases, the formable ranges become narrow. Therefore, the forming temperature must be controlled within a certain range to avoid bad forming results. Forming at too low or too high temperatures may lead to bad forming results. The correlation function of forming temperature with respect to blank diameter and thickness can be calculated from the above figures. When the drawing ratio is between 1.72 and 1.8, the blank thickness is 1.5 to 2 mm. The minimum and maximum forming temperatures can be expressed by the following:

(8) $$T_{min}=20+\left(LDR-1.72\right)\cdot \frac{120}{0.08}$$

and

(9) $$T_{max}=450-\left(LDR-1.72\right)\cdot \frac{40}{0.08},$$

respectively, where LDR (limiting drawing ratio) is the draw ratio of the drawn cup [19]. When the draw ratio is between 1.72 and 1.8, and the thickness is between 1 mm and 1.5 mm, the minimum and maximum forming temperatures are expressed by the following:

(10) $$T_{min}=20+\left(LDR-1.72\right)\cdot \frac{120}{0.08}+\left(1.5-t\right)\cdot \frac{40}{0.5}$$

and

(11) $$T_{max}=450-\left(LDR-1.72\right)\cdot \frac{40}{0.08}-\left(1.5-t\right)\cdot \frac{80}{0.5},$$

respectively, where *t* is the blank thickness. If the draw ratio is 1.8 to 1.9 and the thickness is 1.5 to 2 mm. The minimum and maximum forming temperatures are expressed by the following:

(12) $$T_{min}=140+\left(LDR-1.8\right)\cdot \frac{40}{0.1}$$

and

(13) $$T_{max}=410-\left(t-1.5\right)\cdot \frac{50}{0.5},$$

respectively. If the draw ratio is 1.8 to 1.9 and the thickness is 1 to 1.5 mm, the minimum and maximum forming temperatures can be expressed by the following:

(14) $$T_{min}=140+\left(1.5-t\right)\cdot \frac{40}{0.5}$$

and

(15) $$T_{max}=410-\left(1.5-t\right)\cdot \frac{80}{0.5},$$

respectively. It can be known from the above formable ranges and related functions under the same blank thickness, materials with smaller blank diameters have advantages in forming compared to materials with larger blank diameters. Different blank thicknesses affect the formable range differently. The suitable blank thickness is inside the formable range. A thinner blank may not withstand the material’s stretching during deep drawing and make cracks occur. A thicker blank may be difficult to bend, thus, breakage may occur. Therefore, it is important to control the forming parameter appropriately in the deep drawing process of aluminum alloy A7075.

## 4. Deep Drawing Experiments

### 4.1. Experimental Procedures

**Figure 15:**
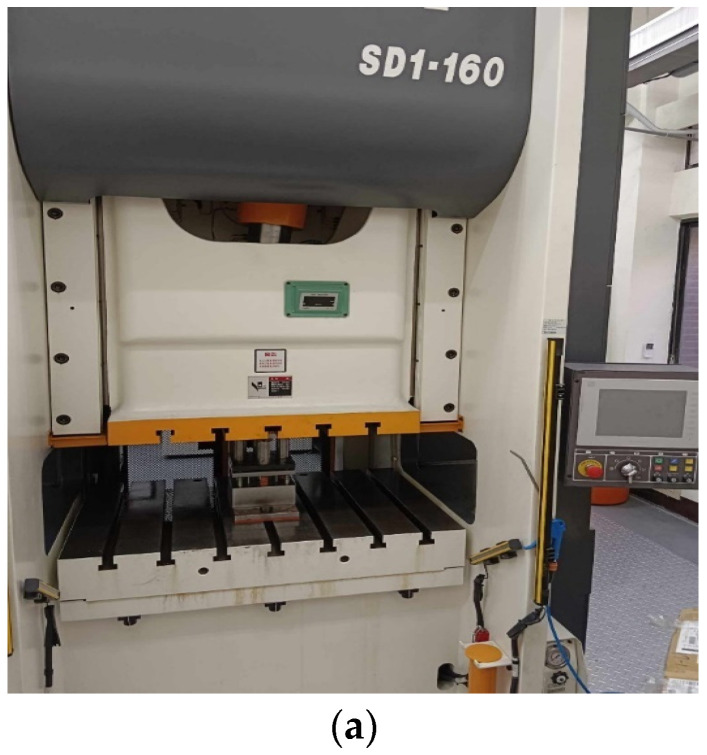

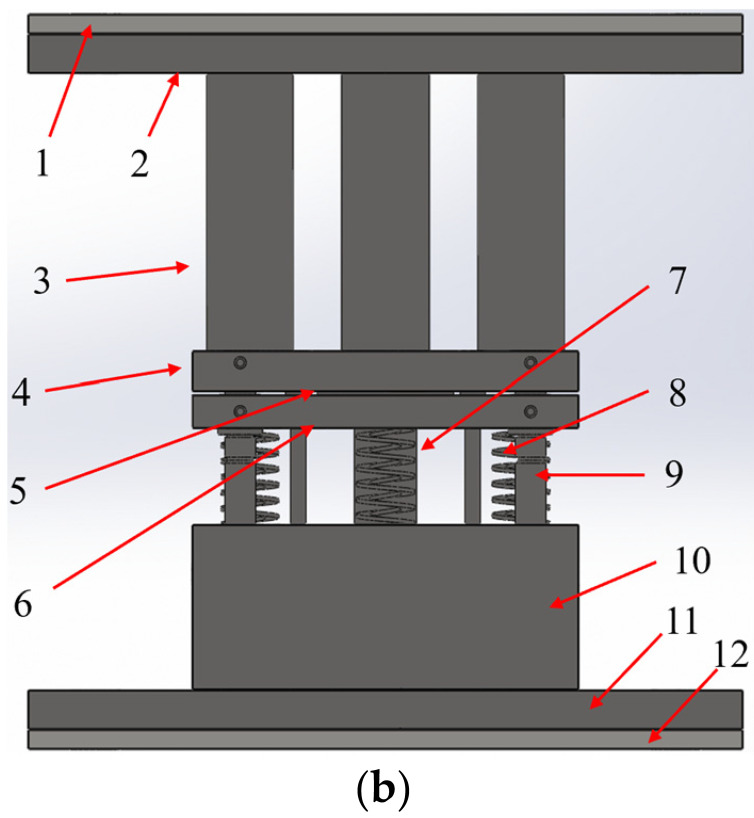
The servo press machine and the deep drawing die set for the experimental procedures. (**a**) Front appearance of servo press machine. (**b**) Assembly drawing of the deep drawing die set.

To validate the finite element modeling and prediction functions proposed, a serious of deep drawing experiments were conducted. A SEYI SD1-160 servo press was used to conduct the deep drawing experiments of aluminum alloy A7075. The SEYI SDI-160 servo press was manufactured by Shieh Yih machinery industry Co., LTD. in Taoyuan, Taiwan. The servo press can control the movement of the upper die precisely. It is equipped with a monitor to show the load variations and punch displacements in real-time. This function makes the user easily set the forming parameters and operate this machine. The appearance of the servo press is shown in Figure 15a and the assembly drawing of the die set for deep drawing processes is shown in Figure 15b. The components in the die set are given in Table 7. The die (no. 4) was designed to be positioned above the punch (no. 7); thus, the relative movement of the punch was opposite to that shown in Figure 1. The blank holder (no. 6) is pressed by four springs (no. 8) with an initial force of 448 N. As the punch moves forward to form the blank (no. 5), the pressing force provided by the springs increases and reaches 2.0 KN at the end of the stroke. Before forming, the workpieces of blank A7075 (no. 5) were heated to a set temperature with a heater, and the punch (no. 7) and die (no. 4) were also heated to the same temperature with a gas torch. Two heat shields were used to prevent heat transfer from the die or punch to the press machine, which may make the machine not function well. After deep drawing experiments, the cup product was taken out of the die set. Its dimensions were measured and some defects such as necking or fracture were checked.

The blank material is aluminum alloy A7075 with a thickness of 2.0 mm. Four kinds of circular blank diameters of 55, 58, 61, and 65 mm were prepared for the deep drawing experiments. The forming temperatures and punch speeds for the experiments are shown in Table 8.

### 4.2. Experimental Results

The experimental results with variations of forming temperatures and punch speeds are shown in Table 9. The initial blank diameter is 55 mm. There is a slightly uneven thickness distribution occurring at the cup rims at lower punch speeds. However, generally, the blanks were formed into circular cups successfully. The forming parameters used in the experiments were inputted into the prediction model to predict the forming results. Table 10 shows the predicted forming results for the blank diameter of 55 mm. Clearly, the predicted results are consistent with the actual experimental results.

The experimental results of the drawn products for a blank diameter of 65 mm are shown in Table 11. Clearly, the drawn products are failures. All the cups are broken under all conditions, which means aluminum alloy A7075 is difficult for deep drawing with a larger drawing ratio. The forming parameters used in the experiments with a blank diameter of 65 mm were inputted into the prediction model to predict the forming results. The prediction results are shown in Table 12. The predicted results show the forming results are failures, which is consistent with the actual experimental results. From the comparisons, it can be said that the prediction models proposed by this study can predict deep drawing results of aluminum alloy A7075 rapidly and effectively.

### 4.3. Comparisons between Experimental and Predicted Results

**Figure 16:**
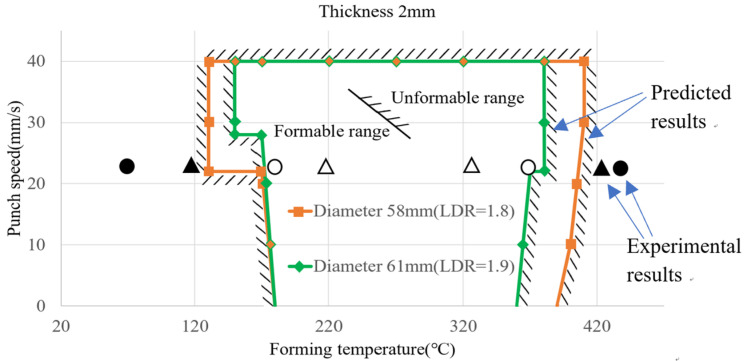
Experimental results and predicted formable regions for different blank diameters and forming temperatures.

The above experimental results have verified the correctness of the prediction model for blank diameters of 58 and 65 mm. Some more experiments were conducted to verify the correctness of the formable regions obtained by the prediction model. The forming conditions and experimental results for verification of predicted formable regions are shown in Table 13. The product appearance for each case is also shown in Table 13. The experimental results and predicted formable regions for different blank diameters and forming temperatures are shown in Figure 16. The inner space is the predicted safe region between forming temperature and punch speed. The outer space with hatched lines is the predicted failure region. Clearly, the predicted formable regions are affected significantly by the forming temperature. The formable region for a blank diameter of 58 mm (drawing ratio 1.8) is larger slightly than that for a blank diameter of 61 mm (drawing ratio 1.9). Symbols Δ and o denote experimental results of sound products for blank diameters of 58 and 61 mm, respectively. Symbols ▲ and ● denote experimental failure results for blank diameters of 58 and 61 mm, respectively. From Figure 16, it is clear that the successful experimental results were located within formable regions, while the failed experimental results were located outside the unformable regions.

From the product appearance shown in the last column of Table 13, it is known that there are some small cracks at the cup rims or uneven thickness distribution on the cup wall for the cases of D = 58 mm, T = 70 °C and D = 61 mm, T = 120 °C. That is, some defects in the drawn cups probably occur at too low temperatures. Bad forming results were found for the cases of D = 58 mm, T = 450 °C and D = 61 mm, T = 420 °C. That is, distortion or fracture at the cup bottom probably occurs at too high temperatures. The experimental results are consistent with the predicted formable regions, which validates the effectiveness of the prediction model by the ML approach.

## 5. Conclusions

This paper investigated the feasibility of applying the ML approach to propose a model to predict the formability of aluminum alloy A7075 in the deep drawing process. At first, a database from the finite element simulation results with various forming parameters was collected. Then, ML classifiers were used for learning and a prediction model was established. A dataset for formable regions of the parameters was constructed from ML training and verification. Some experiments of deep drawing of aluminum alloy A7075 were conducted and the experimental results were compared with the predicted formable regions. The experimental results were consistent with the predicted formable regions and the effectiveness of the prediction model by ML approach was verified. Using this ML approach can save simulation time and shorten the development cycles of new products or processes.

## Figures and Tables

**Table 1:** Geometric and forming parameters used in deep drawing simulations.

Variable	Description	Dimension
$R_{p}$	Punch diameter (mm)	16
$v_{0}$	Punch speed (mm/s)	12.08
$r_{p}$	Punch fillet radius (mm)	5
P	Compression force (N)	Stroke function
$T_{0}$	Forming temperature (°C)	250
$t_{l}$	Blank thickness (mm)	2
$D_{k}$	Blank diameter (mm)	65
$r_{d}$	Die fillet radius (mm)	5
$d_{0}$	Gap between punch and die (mm)	2.2
$D_{d}$	Die inner diameter (mm)	36.4

**Table 2:** Material properties of A7075 used in finite element simulations.

Material Parameters	Values and Unit
Elastic modulus	71,700 (MPa)
Poisson’s ratio	0.33
Thermal expansion coefficients	2.2 $\times 10^{-5}(\frac{1}{{}^{\circ}C})$
Thermal conductivity	41.7 $\left(\frac{N}{sec\times K}\right)$
Heat capacity	0.96$\left(\frac{J}{g\times {}^{\circ}C}\right)$
Mass density	2.81 $\times 10^{-5}(\frac{kg}{{mm}^{3}})$
Yield criterion	von Mises
Failure criteria	Normalized C&L [16]
Friction coefficient	0.1
Heat transfer coefficient	145 W/(m × K)

**Table 3:** Critical damage values of A7075 at different forming temperatures.

Forming Temperature (°C)	Critical Fracture Value
250	0.35
275	0.345
300	0.34
325	0.335
350	0.33
375	0.315
400	0.3
425	0.285
450	0.27

**Table 4:** The level setting for four forming parameters.

Parameters	Level Setting	Number of Levels
Forming temperature (°C)	50, 100, 150, 200, 225, 250, 275, 300, 325, 350, 375, 400, 425, 450	13
Average punch speed (mm/s)	6.0, 12.1, 18.1, 24.2, 30.2, 36.3	6
Blank diameter (mm)	51, 54, 57.5, 65	4
Blank thickness (mm)	0.5, 1.0, 1.5, 2.0, 2.5	5

**Table 5:** Parts of input dataset for CSV file.

Thickness	Diameter	Temperature	Speed	Forming
1.5	51	250	6.0	1
1.5	51	250	12.1	1
1.5	51	250	18.1	1
1.5	51	250	24.2	1
1.5	51	250	30.2	1
1.5	51	250	36.3	1
1.5	51	250	6.0	1
1.5	51	275	12.1	1
1.5	51	275	18.1	1

**Table 6:** Integration of various classifier evaluation indicators.

Model Name	Accuracy	Precision	Recall	F1 Score	AUC
Logistic regression	0.79	0.88	0.69	0.77	0.94
KNN	0.8	0.88	0.71	0.78	0.87
SVM linear	0.75	0.83	0.66	0.73	0.87
SVM Poly	0.73	0.66	1	0.79	0.85
SVM RBF	0.73	0.7	0.85	0.76	0.84
SVM sigmoid	0.33	0.35	0.34	0.34	0.3
Decision tree	0.9	0.84	0.98	0.9	0.96
Random forest	0.98	0.98	0.98	0.98	1
XGBoost	0.98	0.98	0.98	0.98	1
AdaBoost	0.98	1	0.97	0.98	1
Stacking	0.98	1	0.97	0.98	1
LGBM	1	1	1	1	1

**Table 7:** Components in the deep drawing experimental die set.

no.	Components	no.	Components
1	Upper heat shield	7	Punch
2	Top plate	8	Spring
3	Die bracket	9	Guide post
4	Die	10	Punch fixture block
5	Blank	11	Lower heat shield
6	Blank holder	12	Bottom plate

**Table 8:** Forming parameters for deep drawing experiments.

Forming Parameters	Values
Forming temperature${\;T}_{i}\;({}^{\circ}C)$	250, 350, 450
Punch speed $v_{j}$ $\left(\frac{mm}{s}\right)$	12.1, 24.2, 36.3
Blank diameter $D_{k}\;$(mm)	55, 58, 61, 65
Blank thickness $t_{l}\;(mm)$	2.0

**Table 9:** Experimental results of the drawn products with a blank diameter of 55 mm.

Speed	12.08 mm/s	24.16 mm/s	36.25 mm/s
Temperature			
250 °C	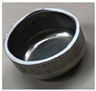	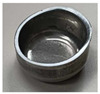	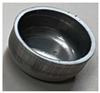
350 °C	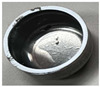	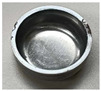	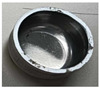
450 °C	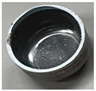	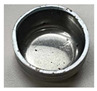	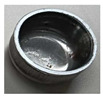

**Table 10:** Prediction results for a blank diameter of 55 mm.

Blank Thickness (mm)	Blank Diameter (mm)	Forming Temperature (°C)	Punch Speed (mm/s)	Prediction
2	55	250	12.1	O
2	55	250	24.2	O
2	55	250	36.3	O
2	55	350	12.1	O
2	55	350	24.2	O
2	55	350	36.3	O
2	55	450	12.1	O
2	55	450	24.2	O
2	55	450	36.3	O
O: Sound products				

**Table 11:** Experimental results of drawn products with a blank diameter of 65 mm.

Speed	12.08 mm/s	24.16 mm/s	36.25 mm/s
Temperature			
250 °C	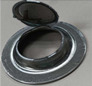	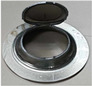	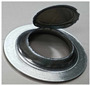
350 °C	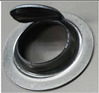	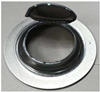	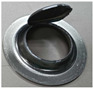
450 °C	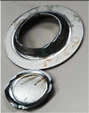	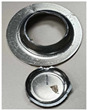	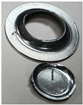

**Table 12:** Prediction results for a blank diameter of 65 mm.

Thickness	Diameter	Temperature	Speed	Predict
2	65	350	12.1	X
2	65	350	24.2	X
2	65	350	36.3	X
2	65	350	12.1	X
2	65	350	24.2	X
2	65	350	36.3	X
2	65	350	12.1	X
2	65	350	24.2	X
2	65	350	36.3	X
X: not ok for products				

**Table 13:** Forming conditions and experimental results for verification of formable regions.

Thickness t (mm)	Diameter D (mm)	Temperature T (°C)	Punch Speed (mm/s)	Experimental Results	Product Appearance
2	58	70	24.1	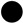	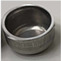
2	58	170	24.1	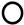	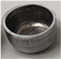
2	58	370	24.1	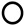	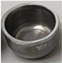
2	58	450	24.1	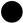	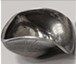
2	61	120	24.1	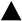	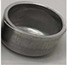
2	61	200	24.1	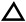	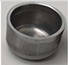
2	61	320	24.1	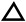	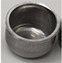
2	61	420	24.1	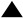	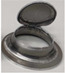

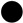
 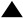: Failure; 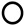 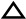: Successful.

## Data Availability

Data are contained within the article.
